# The Hospital. Nursing Section

**Published:** 1905-01-14

**Authors:** 


### The Hospital.

### Nursing Section.

Contributions for this Section of "THE HOSPITAL" should be addressed to the EDITOR, "THE HOSPITAL," NURSING SECTION, 28 & 29 Southampton Street, Strand, London, W.C.

No. 955.—VOL. XXXVII. *SATURDAY, JANUARY* 14, 1905.

### Notes on News from the Nursing World.

### THE KING AND QUEEN AT DEVONSHIRE HOSPITAL, BUXTON.

ON Saturday last, the Devonshire Hospital, Buxton, was honoured with a visit from the King and Queen, who were accompanied by the Duke and Duchess of Devonshire, and several members of the house party, all motoring over from Chatsworth House. The hospital, which stands high, and was once the stables of the seventh Duke of Devonshire, has a famous dome covering an area of half an acre. This allows in wet and cold weather ample exercising ground for the patients to get about in wheeled chairs, walking machines, etc. On the arrival of the King and Queen, they were met at the entrance of the hospital by the Duke of Devonshire, as the hospital's patron. A group of patients drawn up in the colonnade running round the dome, at a signal from the secretary, sang "God Save the King," accompanied by one of the nurses. Their Majesties then walked to the centre of the dome in order to examine the statue of the seventh Duke of Devonshire, and to hear the echo, which is a very good one. They next proceeded to make an inspection of the wards and day-rooms on the ground floor, the King being escorted by his host and Dr. Turner, the principal medical officer, and the Queen, accompanied by the Duchess and Miss Hyland, the matron. Their Majesties were much interested in the patients, many of whom are greatly crippled by rheumatism, the Queen in her gracious manner wishing them a good recovery. On seeing the wooden table in the operating theatre the King remarked that it was not up to date, and later conveyed to the principal medical officer his intention of presenting a glass one, an offer which was most gratefully accepted. In addition to the medical treatment the patients have massage, in which the nurses are trained during their second year. The baths and pump-room for the patients are just outside the hospital grounds, and the patients unable to walk are conveyed to and fro in bath-chairs.

### JAPANESE NURSING IN PORT ARTHUR.

THERE is no doubt that the presence of many thousands of sick and wounded in the hospitals at Port Arthur will put the Japanese Red Cross Society on its mettle, especially as it is declared to be impossible to move the majority. But it is also certain that even this tremendous demand upon its resources had been anticipated by the far-seeing managers of the organisation. Like us, the Japanese have an Army Nursing Reserve, only it is on a larger scale than ours, owing to the fact that the nurses, after their training, are allowed to return to their ordinary avocation and pursue it until their services are required by the Red Cross Society. Assuming that the Japanese Army nursing system emerges triumphant from the severe test to which it is now exposed, other nations will probably pay it the compliment of imitation.

### PROGRESS OF NURSING IN COREA.

WE understand that a trained nurse and a lady dispenser are going out to Chemulpo Hospital during the spring. Mrs. Weir, the wife of Dr. Weir, who took charge at Chemulpo in the summer, is a Gold Medallist of St. Bartholomew's, and the hospital has been completed and greatly improved. The out-patient department is a very important feature. The nursing at the first English hospital opened in Corea, at Seoul, was for some time done by Kilburn sisters, but they have been gradually replaced by trained nurses. There was a woman's department at Seoul for several years, but it was closed when the war between Russia and Japan broke out, owing to the return to England of the lady in charge. It is hoped that a ward for women may shortly be opened at Chemulpo. The keen and growing appreciation shown by the natives for treatment, especially surgical, suggests that there will be considerable scope in Corea for nurses later on.

### NO CUBICLES AT GRIMSBY HOSPITAL.

WE are pleased to learn from the chairman of the Grimsby and District Hospital that, having read our remarks deprecating the proposal to mar the new Home by providing cubicles for the nurses, instead of single bedrooms, he considers our conclusions unanswerable, and intends to lay our ideas before the committee, "with a view to having the plans altered at once." After this expression of opinion on the part of the chairman, we may assume that single bedrooms will be provided in the home, to the great advantage of all concerned.

### POOR-LAW INFIRMARIES AND THE TRAINING OF MIDWIVES.

AN important circular has been issued by the Bradford Guardians on the subject of the non-recognition by the Central Midwives Board of Poor-law Infirmaries as training schools for midwives. It is pointed out in the circular that the Central Midwives Board have refused to recognise Bradford Poor-law Infirmary Training School in spite of the fact that for some time it has turned out an average of 11 nurses annually who have passed the examinations and obtained the certificates of the London Obstetrical Society. Bradford, of course, is not an isolated case. Several similar applications have so far been unsuccessful. The memorial which the Bradford Guardians have sent to the Central Midwives Board should not therefore stand alone if the authorities of the principal Poor-law Infirmary Training Schools wish to secure recognition from the Board. We agree with the contention that in such schools midwifery can be taught in the most satisfactory manner, both theoretically and practically; and in these circumstances it would not be fair to shut them out, providing, of course, that they conform to the regulations.

### A CRISIS AT PLYMOUTH INFIRMARY.

Matters have reached a climax at Plymouth Workhouse Infirmary. Exasperated at the frequency of the changes in the nursing staff, the Hospital Committee recently came to the conclusion that a change of management is essential, and in order to secure this they recommended the guardians to call upon the superintendent nurse to relinquish her post. The recommendation was adopted, and the superintendent nurse was requested to send in her resignation by December 21st. She declined to comply with the request, and in her letter said that she was greatly grieved to receive such a communication, because she was not aware that there was the slightest complaint either from the board or the medical officer against her. On the contrary, the guardians had expressed their entire satisfaction with the manner in which her duties had been performed. As such a resolution might do her incalculable harm in her profession, she requested that the grounds on which the guardians had founded their resolution should be furnished her in detail, so that she might have an opportunity of replying to the same. The matter was then referred to the Hospital Committee, who considered it on Friday last, and instructed the clerk to inform the superintendent nurse that there was no specific complaint against her, but that the friction rendered her resignation desirable. We think that the reasonable request of the superintendent nurse should be complied with.

### THE FEES OF A MATERNITY AND NURSING AID SOCIETY.

Our attention has been called to the conditions on which the Bristol Cottage Hospital Maternity and Nursing Aid Society receive probationers. An applicant for terms was informed that "a first-class general and midwifery training" is given at the hospital in 12 months for a fee of "from £30 to £45." It was also stated that at the end of that period the authorities of the hospital are able to get a nurse "a very good post." There are different ideas as to what constitutes a very good post, but it is obvious that a year's training at the Bristol Cottage Hospital will not enable a nurse to obtain any appointment either at a general hospital or a Poor-law infirmary which is a recognised school of training. On the other hand, "from £30 to £45" is a ridiculously high fee to pay to be qualified to act as a cottage nurse.

### NO NIGHT NURSE FOR NORTHALLERTON INFIRMARY.

The Northallerton guardians are incorrigible. In spi e of the strong protest of some of the members, who affirmed that a night attendant in the Infirmary was necessary for the old people, and that the very least the guardians could do for them was to make them as comfortable as possible, the majority voted against a proposal to engage one. The view expressed by the Chairman was that there were inmates of the Infirmary who would awaken the nurse in case of necessity. Evidently the idea that a nurse cannot reasonably be required to perform day and night duty did not occur to him, and the remark also shows a strange lack of consideration for the inmates, which we should not have expected from a clergyman. The refusal to appoint a night attendant was, of course, urged on the ground of economy, but no anxiety for the ratepayers' pockets can justify insufficient care either of the sick, the infirm, or the aged.

### AN INJUSTICE TO AN INSTITUTION.

An extraordinary attempt has been made to impute blame to the Coventry Nursing Institution owing to the conduct of a nurse who was formerly connected with it being questioned elsewhere. The Coventry institution has two branches, and the nurse was attached not to the district, but to the private branch. Her association with it, the authorities affirm, was perfectly satisfactory throughout, and we certainly do not condemn them because in the hour of her need they afforded her assistance. We cannot understand the attitude of those persons who, for such a reason, speak indifferently of an organisation which has for years been much valued in the town.

### NURSES AND AMERICAN FICTION.

At the annual New Year's Day meeting between the managers of the Glasgow Royal Infirmary and the nurses, the Lord Provost, who prefaced an extremely interesting speech by telling the staff "that the plans of the magnificent new building were under the consideration of Sir Henry Burdett and Dr. Donald McIntosh, two of the greatest authorities on hospital construction and management in the country," dwelt on the stimulus which could be had from reading. He particularly called the attention of nurses to some of the charming works of fiction from America, which he said, came with a freshness and delight equal to, and yet different from, their own. He commended to them several examples of this school, notably "Lavender and Old Lace," his eulogium eliciting from Sir Samuel Chisholm the humourous suggestion that the treasurer should write to the publishers of that book and ask for a cheque for £500.

### THE MOULSFORD CASE.

In spite of the verdict of the jury in respect to the young woman who died in the Bucks County Asylum, the county justices at Wallingford committed the nurse who assaulted an inmate on a charge of manslaughter. The verdict of the coroner's jury, it will be recollected, was that the deceased died from hæmorrhage of the brain caused by excitement while being removed to the lavatory. The medical superintendent of the asylum, however, stated at the inquiry before the magistrates that the most probable cause of the hæmorrhage was the blow, and in the face of this statement, we do not see how the magistrates could do otherwise than send the case for trial. The accused will, we hope, be provided with adequate legal assistance.

### THE HOSPITAL FOR MOTHERS AND BABIES.

It has been decided by the Council for the Promotion of the Higher Training of Midwives to open, in April, a maternity hospital in the houses, 63 and 64 Upper Wood Street, Woolwich, which shall be used as a training school for district midwives. This, of course, is only a portion of the scheme presented by

Miss Alice Gregory and her friends, of which full details have been given in our columns. But the carrying out of that portion of it which has reference to a general hospital, being for the time impracticable, only the Maternity Hospital will be proceeded with at present. It will at the outset consist of 12 beds, will be known as the Hospital for Mothers and Babies, and the nurses taking their maternity training will have received at least a year's general training elsewhere. A district midwifery practice will be attached to the institution. Princess Christian has promised to perform the opening ceremony.

### THE MIDWIVES ACT IN RURAL DISTRICTS.

A meeting, under the auspices of the Rural Midwives Association, will be held, by permission of Mrs. Murray, at 50 Albemarle Street, W., on Wednesday, January 25th, at 2.30 p.m., to consider the best means of carrying out the Midwives Act in rural districts. The questions of supervision, inspection, the best type of midwife, the financial aspect, and various difficulties will be fully discussed. Any practically interested in the subject who desire invitations should communicate with the Secretary of the Rural Midwives Association, 47 Victoria Street, S.W.

### NURSES' QUARTERS DESTROYED BY FIRE.

A fire occurred at Buckinghamshire County Asylum on Monday morning. The alarm was given by a night nurse. She was going to bed at a quarter past nine when she noticed that the ceiling of the nurses' department in the women's wing was on fire. She immediately gave the alarm, and a messenger was despatched from Stone to Aylesbury for the brigade. Meanwhile the attendants at the asylum had prevented the fire from spreading, and the brigade on their arrival speedily got it under. The roof and ceiling of the old female wing, now used as the nurses' apartments, were destroyed, but the fire did not extend to the main building, and not only were none of the patients injured, but no inconvenience was caused to them. The damage is estimated at over £1,000, but if the night nurse who gave the alarm had been an unobservant person the consequences of the outbreak might have been disastrous.

### CHARGE OF IMPOSING UPON MIDWIVES.

A man named William Morgan was on Friday last committed for trial on the charge of obtaining money by false pretences. It is alleged that the prisoner, representing himself to be an inspector under the Midwives Act, had called on midwives alleging that he was making an official register showing the qualifications of ladies. He is accused of receiving fees for placing the midwives under "Class A" in the supposed register, this class showing the best qualifications, and for preparing certificates. With regard to these charges, it was alleged by witnesses in certain localities, that Morgan said "he had been sent to see how many midwives were required for each parish." As the case is *sub judice*, we, of course, make no comment on the nature of the accusations; but it will be recollected that on October 29th we drew attention to an enterprise described as the "Medical Register," which nurses were being invited to avail themselves of in order to avoid getting thrown out of work. We suggested at the time that this was a swindle, not only because nurses are not registered, but also because we had ascertained that at the address given in Fleet Street, nothing whatever was known of either J. Howard and Co., the supposed managers of the "Medical Register," or of W. Morgan, who signed receipts for 3s. in their name. It will be observed that the person who is asserted to have imposed, or tried to impose, upon some 60 midwives, has the same name as the person who acted on behalf of "J. Howard and Co.," and that his *modus operandi* bears a suspicious resemblance to that of the individual associated with the "Medical Register."

### CHELSEA HOSPITAL FOR WOMEN.

The Christmas festivities at Chelsea Hospital for Women terminated with a "treat" arranged by the ladies' committee, consisting of a conjuring entertainment, a tea, and a Christmas tree. Fortunately, a very large percentage of the patients were able to leave their beds, and together with the nurses and numerous visitors they greatly enjoyed the clever conjuring of Mr. Maurice Victor, of the Egyptian Hall. The entertainment took place in the Dyer-Edwards Ward, which was appropriately decorated. All the wards were adorned with flowers, evergreens, and fairy lamps. After an interval for tea, the company withdrew into another ward, where the handsome Christmas tree, the gift of Dr. and Mrs. Fenton, lighted by electricity and laden with presents, was much admired. A distribution of presents to the patients and nursing staff followed.

### HOSPITAL OF ST. JOHN AND ST. ELIZABETH.

The Christmas entertainment at the Hospital of St. John and St. Elizabeth took place in the children's ward on Tuesday when a large number of visitors were present. The beautiful Christmas tree, given by Lady Clifford, of Chudleigh, was laden with presents for all the patients, and lighted by red-shaded electric lamps. The ward decorations were exceptionally good, owing mainly to the care bestowed upon them by the electrician of the hospital. For weeks past he had been working at the decorations and superintending the labours of the ward children who made the pretty blue and white paper festoons with which the ceiling of the ward was draped. These paper decorations were interwoven with garlands of artificial flowers and fruit which were all lighted by electricity. The shrines at the end of the ward were also illuminated. After the tree had been dismantled the visitors carried gifts to those patients who were unable to be moved in to the entertainment.

### SHORT ITEMS.

The Queen has sent an annual subscription of £5 to Queen Charlotte's Hospital, and the Princess of Wales has also sent a contribution of £5.—The Princess of Wales has contributed ten guineas to the Cornwall County Nursing Fund.—The annual treat to the crippled children at the City Orthopædic Hospital took place on Tuesday evening in the presence of many members of the board of management and medical staff. The decorations and arrangements were organised and carried out by the ladies' committee who specially collected for the entertainment.

### The Nursing Outlook.

"From magnanimity, all fear above;
From nobler recompense, above applause,
Which owes to man's short outlook all its charm."

### MISS MONK ON REGISTRATION.

"It surely behoves all who have the interests of nurses and nursing work at heart, and who are striving so earnestly for their improvement, to endeavour to come to some unanimous decision that would once and for all place them in their true position, and guard them from all future misconception and disrepute, a decision that would restore the nurse and her work in the eyes of the world to the noble position they held when the refined and beloved Florence Nightingale was still strong enough to be their leader, when nurses were held to be the pick of their sex, and were supposed to be above feminine jealousies and party politics."
MISS MONK in *The Monthly Review*, January, 1905.

It is a noble ambition to place the nurse in her true position, and guard her from all future misconception and disrepute. Why is it that misconception and disrepute so dog the footsteps of the modern nurse in Great Britain to-day? There are many reasons. They include the ambitions of a small clique who unceasingly labour to secure control over the nurses of this country, and who are known as the "stage army"; the failure to exhibit public spirit by some managers of the chief nurse training-schools, some of whom farm their nurses for their own profit; the fact that practically almost any woman can obtain employment as a nurse, although she may never have been properly taught nursing, her brief experience being confined to some special cottage or fever hospital. As to the latter reason, a very careful consideration of the actual facts convinces us that the public at the present time are served for the greater part by the untrained rather than by the trained nurse.

This is a state of affairs which demands a prompt and practical remedy. Miss Monk, whose article should be read, sets herself to show that the State registration of nurses is not a panacea, and never can be, for the existing evils. Registration, "can never guarantee to the sick or the medical profession that they have the right nurse, that is, one not only technically capable, but also able to take her proper place in the household of her patient, where she should naturally be a comfort and a help." If registration cannot give the public and the profession this guarantee, and who can maintain that it will, State registration is revealed at the best to be a mere register of names and qualifications, the value of which, so far as the older nurses are concerned, must decrease, from the employer's point of view, every year. Our position is and always has been that we have no objection to the State registration of nurses, for the mere keeping of a register can do no harm providing the public can be made to realise its precise limitations. Otherwise it may subject them to risks of even greater evils than they at present run from attendance by incompetent nurses in the hour of sickness. A general register of nurses who have undergone a definite and uniform training, and who have shown by examination an average capacity and knowledge, cannot be objectionable in itself, and has never, we suppose, been objected to as such. When however the "stage army" claims that if Parliament grants State registration the whole nursing position will be cleared, and the public and the profession will be afforded the guarantees which are essential to their protection, every one of knowledge, authority and character is in duty bound to oppose such a view as mere humbug.

We have striven for years to educate the public to apprehend and appreciate the transparent falsehood of claims which have been so persistently put forward in favour of State registration. We hope therefore we shall hear no more of the humbug crusade which is mainly responsible for the confusion into which nursing matters have drifted in this country, and that State registration will be given the open door and back seat which its limited possibilities entitle it to claim. The House of Commons may consider it has far more important matters to attend to, than the State registration of nurses, when the members appreciate, at its true value, the limited effects which such a system must have in redressing the existing evils of which the public justly complain.

Miss Monk states that the necessary guarantees should be obtainable by the medical man or the employer from those who supply the nurse. This is a good point to make, but before a guarantee of this kind can be obtained, Parliament must insist upon the licensing and registration of surgical and medical homes, private nursing associations, hospitals, and agencies of all kinds which supply nurses to the public. What is really urgently required is the establishment, with the co-operation of some of the more intelligent and public-spirited nurse training schools, and teachers and instructors of probationers, of a Central Council of Nursing Education, properly constituted by charter or otherwise on an authoritative basis. This central council of nursing education would throw its examinations open to all nurses wherever trained, decide upon a definite course of consecutive training, and issue a standard certificate representing the average requirements which in their judgment will afford a reasonable guarantee to the public. The form of certificate should embrace evidence as to the moral and personal qualities of each nurse, certified by those who have had her in charge during her period of probation. Under some such system the public could be guaranteed that the nurse supplied to them had started on her career with the necessary qualifications. The Central Council of Nursing Education might readily and usefully organise a system which should provide for the educational requirements of post-graduate nurses, whilst it afforded guarantees that all those who pass through its hands must continue to be worthy members of an honourable profession for honourable women, or forfeit their certificates.

### Lectures upon the Nursing of Infectious Diseases.

By F. J. WOOLLACOTT, M.A., M.D., B.S.Oxon, D.P.H., Senior Assistant Medical Officer, Park Hospital, Metropolitan Asylums' Board, Hither Green.

### LECTURE XII.—MEASLES.

*Measles* or *Morbilli* is an acute infectious disease characterised by a peculiar blotchy eruption on the skin. Even more than scarlet fever and diphtheria it is a disease of childhood, and is particularly dangerous in the first years of life. It is most prevalent during the early summer and winter, the greatest mortality occurring in the months of June and December. The infection usually spreads directly from person to person, and occasionally by the agency of clothes or other articles that have come in contact with the sick. Unlike scarlet fever it has never been known to result from the consumption of infected milk, and there is no evidence that it is due to impure water. Outbreaks of the disease vary considerably in severity and while in some nearly every case is of a mild and comparatively harmless type, in others there is frequently grave danger, either from the intensity of the symptoms or the appearance of complications. Up to the present no systematic attempt has been made to isolate measles in hospital. It is unfortunate that there are so many difficulties in the way, for there is no doubt that the removal of patients from insanitary surroundings together with careful nursing would result in the saving of many lives.

The incubation period is usually about ten days, and after a person is exposed to infection three weeks or at least a fortnight must elapse before we can be certain that he has escaped.

As a rule the earliest symptoms are those of a severe cold in the head, the eyes, nose and respiratory passages all being involved. The conjunctival mucous membrane is injected, the eyes water and cannot bear exposure to the light. From the nose a thin discharge runs, and sneezing is rarely absent. The throat feels a little sore and there is usually a frequent dry cough. The fauces are red and perhaps swollen, but never to the same extent as in scarlet fever. Should the inflammation extend to the larynx the cough becomes croupy, and the breathing may become obstructed. The temperature is raised from the commencement, but often falls somewhat on the second or third day, to rise again on the appearance of the rash. The patient is fretful and irritable or occasionally drowsy. Convulsions may occur, and more or less delirium is not uncommon. The presence of these symptoms is always highly suggestive of measles, but certainty may be impossible until the rash shows itself. An additional sign of the utmost value is the appearance on the mucous membrane of the mouth of small spots, named after their discoverers Koplik or Filatow spots. They are frequently visible as early as the second day of the disease and usually persist until the rash is well out, their commonest site being the mucous membrane of the cheeks just opposite the molar teeth. Owing to their small size they are sometimes difficult to see, but with a good light they appear as small white dots, varying in number from three or four to a score or more, and each one surrounded by a red areola. As they have only been observed in measles their presence may be regarded as conclusive evidence of that disease.

The characteristic rash appears on the fourth day of illness. In some cases it is preceded by various transient skin eruptions, the commonest of which are a diffused redness, not unlike scarlet fever, and nettlerash. Usually it is first seen on the forehead or behind the ears, and soon spreads to the rest of the face and then downwards to the trunk and limbs. It involves the whole surface of the body and does not, like the rash of scarlet fever, avoid the parts round the mouth. At first it consists of small red papules but these soon enlarge and coalesce one with another to form irregular and slightly raised blotches. The colour is a dull or dusky red, less bright than that of the scarlet fever rash, and often does not disappear entirely on pressure. When the eruption is well developed the appearance and condition of the patient is very typical. The face is puffy and, together with the rest of the body, covered with thickly set and disfiguring blotches. The skin is hot and irritable, and more or less itching may be present, which, together with the cough, sneezing, and watering of the eyes, may be a source of much discomfort. The temperature is raised, often to 104° F. or more, and in consequence the patient is restless and unable to sleep. Appetite is almost lost, but as a rule there is considerable thirst.

In the great majority of cases the rash soon begins to fade, the face clearing first. It often leaves a yellowish brown staining of the skin, which may persist for several days. At the same time the condition of the patient rapidly improves, and, in the absence of complications, convalescence is quickly established. The temperature falls, the catarrhal symptoms disappear, appetite returns, and refreshing sleep is obtained. The fading of the rash is frequently followed by slight desquamation of the skin, of a dry powdery or branny character and very rarely as extensive or complete as the peeling so characteristic of scarlet fever.

Occasionally an attack of measles will deviate considerably from the ordinary type of the disease just described. On the one hand it may be very mild, with but little discomfort, while on the other it may prove of extreme severity quite apart from the presence of complications. Two varieties of the severe or malignant type are met with. In the first of these the onset of the disease is very acute and the patient is rapidly prostrated. The temperature is high and keeps steadily up, the respiration is rapid and the pulse-rate much increased. The rash is usually ill-developed or absent, while delirium and perhaps convulsions make their appearance. The cough is troublesome and more or less cyanosis usually present. Unless improvement quickly takes place the patient sinks into a state of coma and dies. The second variety of the malignant form is characterised by the appearance of hæmorrhages into the skin together with bleeding from the nose, mouth and bowels. It is fortunately very rare. The patients are usually ill-nourished and weakly children and invariably succumb.

In the treatment of measles the same general principles should be observed as in other infectious diseases. The patient should be kept in bed in an airy, well-ventilated room, and an even temperature of 65° F. or a little over should be maintained. In the acute stage the room should be somewhat darkened, as the light is very trying to the inflamed eyes. If there be troublesome and frequent cough relief will be given by using the steam-kettle to moisten the air. It is important that the patient should be screened from draughts, and care must be taken that he does not receive a chill when he is washed. As we shall see, the chief and most dangerous complication of the disease is pneumonia, which may be readily induced if the precautions just mentioned are not observed. During the febrile stage the food should be liquid and consist largely of milk. Water may be given in moderation if asked for, and will probably satisfy the thirst better than anything else. As the symptoms subside the diet should be gradually increased, and in the convalescent stage should be plentiful and nourishing.

If the child be feverish and fretful, tepid or warm sponging will be found of considerable service. It will allay the irritation of the skin, soothe the delirium, and promote sleep.

The discharges from the eyes and nose should be removed by pieces of lint or rag, which must be burnt immediately after use. The eyes should receive special attention. They must be carefully and frequently bathed with some warm lotion, for, if the discharge is allowed to accumulate, or if the child is allowed to rub them to ease the irritation, severe inflammation may follow. Attention should also be paid to the mouth and throat, especially in the more severe cases. Sores are very apt to form on the tongue and on the mucous membrane of the gums and cheeks, and should be prevented as far as possible by the use of antiseptic mouth washes. In some cases it may be necessary to syringe the fauces. Itching of the skin may be relieved by the application of weak carbolic oil.

A few days after the rash has faded and the temperature fallen to normal the patient may be allowed to get up, at first for a short time only each day. The clothing should be warm and he should be kept for some days longer in a warm room and shielded from draughts until convalescence is well established. At the end of three weeks, or less in summer time, he may be allowed to go out if the weather is fine. It is important to observe these precautions for, apart from the question of infection, there is the possibility that complications may supervene some time after the acute period of illness is over. In the case of young infants especially great care should be taken to avoid the risk of chill or undue exposure. Unfortunately there is a tendency among many people to regard measles as only a trifling illness, and but little trouble is taken in the later stages. How erroneous this view is will readily appear if we remember that in this country it causes more deaths than even such a universally-dreaded disease as scarlet fever.

The treatment of the severe and malignant types of the disease is difficult and often unsatisfactory. Our efforts should be directed to reducing the temperature, allaying the delirium and restlessness, and maintaining the strength of the patient as far as possible by suitable nourishment. The cold pack may prove of considerable service, and at times a warm or tepid bath is of benefit. Collapse must be guarded against by the use of stimulants. For delirium or headache it is a good plan to wrap the head in a cloth kept constantly wet with cold or iced water.

The complications of measles and their treatment will be considered in the next lecture.

### Sponging a Fever Patient with Tepid Water.

### EXAMINATION QUESTIONS FOR NURSES.

THE question was as follows:—(1) How would you carry out an order to sponge a fever patient with tepid water? (2) How would you proceed to cold-sponge a similar case?

### FIRST PRIZE.

Before starting to tepid sponge a patient I should give some nourishment, according to orders received from the doctor. For instance, in an ordinary case a few ounces of milk, or if the patient was in an exhausted condition and was ordered a stimulant, I should add to the milk half an ounce of the prescribed stimulant. For instance, four ounces of milk and half an ounce of brandy.

I should then get ready all that I required, a basin of water at a temperature about 100° to allow of its cooling, a bath thermometer, a large sponge or piece of soft flannel, and two or three hot towels. After filling two or more hot-water bottles and placing them near in case patient showed signs of collapse with a hot blanket and stimulant handy. I should also have a jug containing boiling water from which I could add to the water in the basin from time to time to keep the temperature at about 85° to 90°.

I should then proceed with the patient, removing the body-linen and top bed-clothes, placing the body-linen in front of the fire to warm. I should have two, if possible, thin old blankets, kept for the purpose, one to place over patient and the other underneath so that he lies upon it. By having two blankets there is less risk of exposure, as the patient can be better covered than with one only. I should then begin to sponge, first the chest and then, if the patient is able to be turned on to his side, the back, and then the limbs separately, sponging downwards and wetting the sponge frequently, being careful to keep up the temperature of the water by means of the jug, and avoiding wetting the blankets. After sponging each part I should dry it immediately with a warm towel, keeping patient covered with the blanket. Tepid sponging should take from fifteen to twenty minutes, and must be carried out quietly and thoroughly, avoiding bustle and exposure of the patient. When I had finished I should let patient remain in the blankets for about ten minutes, after which I should take his temperature and, if reduced, replace his warmed clothing and bed-linen.

To carry out cold sponging proceed in the same way with the exception that to begin with the water should be at a temperature of 90°; to avoid shock gradually reduce it by means of cold water or, if ordered ice-cold, by pieces of ice until it reaches the required temperature, 65° to 70°. I should also place a hot bottle to patient's feet before starting. The sponging should be carried out in a much shorter time—about ten minutes—and the patient would need careful watching, and should he show any signs of collapse the sponging should be at once stopped, hot blankets and bottles applied, and restoratives given.

"CENTRAL."

### SECOND PRIZE.

When about to commence to sponge a fever patient the exact temperature of the body must first be noted.

For tepid sponging the water prepared should be from 80° to 90° Fahr. Remove all personal clothing from patient and place blankets both under and over him. Place beside the bed all basins, sponges, and towels, or anything that may be required, as under no circumstances should a patient be left during the operation. Commence at the head and sponge downwards, exposing only one limb at a time. When the whole body has been sponged the patient should be wrapped in a warm blanket and left undisturbed for an hour or even longer. The temperature may then be taken again to ascertain how much it has been reduced.

The same precautions should be used in cold sponging as in tepid. It will, however, be found advisable to sponge each limb over with tepid water before applying the cold as it will then cause less shock to the patient.

It is wise to keep a hot bottle to a patient's feet during sponging, as with the feet warm there is less fear of chill, and to a fever patient there is always more or less danger of collapse. When the temperature has been reduced the body must be gently dried and a flannel night-gown put on. After a cold sponge the temperature may fall from one to six degrees, the colder the water the sooner the reaction takes place. Ammonia, Eau-de-Cologne, or vinegar added to the water makes it more cooling by its rapid evaporation.

Sponging can also be done by wringing towels out of cold water, dry enough not to drip, and placing them one after another from the neck downwards. When the feet are reached begin again at the head and renew each in succession, continuing as long as necessary. "HYGEIA."

### STANDARD GOOD THIS MONTH.

The answers this month taken as a whole are very much above the average. "Central" sends an excellent paper she is always a most painstaking and thoughtful candidate. She takes the first prize because, though her answer is not faultless, it is the best one all round that has been sent in. She wisely thinks of giving a small amount of nourishment before the process begins; she has her warm water hotter than is necessary to allow for unavoidable delays at the last moment, and when cold sponging she takes the precaution of placing a hot bottle at once to the patient's feet instead of waiting to do so until there are signs of collapse. She shows common sense and resourcefulness in beginning with warm water at first instead of clapping a cold sponge suddenly on an already enfeebled body; 70° is rather high for a cold sponge, but really extreme cold such as 50° is seldom employed now.

"Hygeia" takes the second prize with a very good paper, but she is a little vague, as when she speaks of "the reaction" after the cold sponging, and she wanders from the point when she enters upon a description of what is more of a "cold pack" than cold sponging. Neither of the prize-winners mentions one point which is important and which is noted by many other candidates whose papers are not on the whole so good, namely, the advisability of paying particular attention to thorough sponging of the axilla, the groin, and inside the thigh.

There is great diversity of opinion as to the exact temperature which constitutes tepid and cold, but authorities do not always agree, and each doctor usually states the exact temperature he wishes employed.

Honourable Mention.

This is gained by "Kent," "Festina Lente," "Simple," and "Thatchhouse." I hope this last name is correct. Nurses sometimes give so much trouble by not taking pains to write legibly. "Festina Lente" and "Kent" both send excellent answers.

Question for January.

Describe how you would arrange constant hot irrigation to an injured leg. N.B. This question was asked in December 1903. The answers were not satisfactory and it is hoped that now more thought will be brought to bear on the subject.

The Examiner.

Rules.

The competition is open to all. Answers must not exceed 500 words, and must be written on one side of the paper only, without divisions, head lines, or marginal notes. The pseudonym, as well as the proper name and address, must be written on the *same* paper, and *not on a separate sheet.* Papers may be sent in for fifteen days only from the day of the publication of the question. All illustrations strictly prohibited. Failure to comply with these rules will disqualify the candidate for competition. Prizes will be awarded for the best two answers. Papers to be sent to "The Editor," with "Examination" written on the left-hand corner of the envelope.

In addition to two prizes honourable mention cards will be awarded to those who have sent in exceptionally good papers.

N.B.—The decision of the Examiner is final, and no correspondence on the subject can be entertained.

Any competitor having gained three prizes within the current year shall be disqualified from taking another until 12 months shall have expired since the first prize was gained.

### A Visit to a Japanese Nursing Home.

BY AN ENGLISH SISTER.

A substantial, two-storied, English-looking building heated by hot-water pipes and lighted by electricity.

The staff consists of a resident house surgeon, a matron, and some eight or ten nurses, according to the number and severity of the cases. A visiting surgeon—a German professor—performs the majority of the major operations. The surgeons wear large white linen sterilised overalls, and the nursing staff white dresses and aprons made English fashion with "Sister Dora" caps.

A new operating theatre has recently been built at the end of a long corridor on the ground floor. Everything looks very up-to-date and aseptic. There is a good sterilising apparatus, white washable walls, tiled floor, and an excellent supply of light.

As to general accommodation, there are about 13 available rooms and two small general wards, each containing six beds. Both rooms and wards have highly polished floors and the former are supplied with rugs.

The furniture is very simple—a Lawson Tait bed, a comfortable wicker chair, several of the upright cane pattern, an invalid bed-table, a small ordinary table, a combination bureau and dressing table, a small triangular washstand and the inevitable screen. Long cane lounges can be had for convalescent patients. The windows have short white curtains and white blinds and even at this time of year, November, on a sunny day the glare is somewhat trying. Most of the rooms have nice framed engravings such as Millet's "Angelus" and the "Sistine Madonna."

A card of simple rules is affixed to each door giving visiting hours, regulation meal times and requesting patients not to have food brought in without permission from the surgeon or matron.

Everything is scrupulously clean, the nurses doing all sweeping and dusting. The floors are wiped over every morning with a disinfectant solution and the dusting is done on the wiping-off principle with a damp cloth. Temperatures and pulses are taken two, four, six, or eight times hourly according to the patient's condition, and charts are kept for the doctor's inspection in the duty room. Oiled paper is largely used in place of rubber sheeting and seems to make a fairly good substitute. All instruments and dressings are sterilised.

The house-surgeon and matron speak and understand English very well, and most of the nurses can understand ordinary wants. They are most courteous, skilful, and attentive, and very gentle and quiet.

The food is excellent in quality and most daintily served. Such nice little trays, covered with snowy cloths—all the little details so complete—pretty thin cups, dainty little butter dishes, with butter quite artistically arranged; plenty of fruit, both raw and cooked.

A very good dispensary is attached, in which outsiders can procure ordinary drugs at a moderate rate and prescriptions are made up.

Altogether, the home struck me as being exceedingly well conducted and it would compare favourably with many English establishments.

### Where to Go.

Messrs. Penberthy, 388 Oxford Street.—Messrs. Penberthy are now holding their winter sale, and they inform us that they are selling at cost, or under, in all departments.

### "The Hospital" Convalescent Fund.

The Honorary Secretary acknowledges with many thanks the receipt of £1 from Nurse Attree and 5s. from Nurse Whyte.

### The Training of Mental Nurses on Hospital Lines.

In the article published last week upon the "Training of Mental Nurses on Hospital Lines in Theory and Practice" the interest taken by Dr. W. F. Menzies, now Medical Superintendent of the Cheddleton Asylum in Staffordshire is appreciated, and there is no doubt that age and maturity are important questions in the selection of asylum nurses.

It may be suggested, however, that he does not place sufficient importance upon one point in the employment of women. There are few women who seriously set before themselves as the object of their lives anything apart from the great end intended by Nature for women, viz., that they should become wives and mothers. To delay engaging women as asylum nurses until the age of 25 is to open a door to many women who have hitherto failed in this as well as other departments of energy. Service in asylums is recognised to be of an exceptional character, and the sooner apprenticeship is commenced in it the better. Many young women are at their best at the age of 21, both in interest, adaptability, and physique, and complaints as to their want of tact and judgment are applicable to any age, and often more so when over 25 years, as by that age pre-conceived ideas and intolerance of singularities have probably become fixed. It is too much, I think, to expect any nurse, after constant attendance upon the insane, to attend lectures, rehearsals, choir practices, and what not in their own time. In the London asylums every nurse has one day off duty in seven, and there is no "evening" duty. Every male nurse has one-and-a-half days off in every seven days. In these asylums, when the time for meals and dressing is deducted, the female nurses are on duty for 69 hours a week, and the male nurses for 63¼ hours. Most administrators think 7.30 A.M. too late for the day staff to commence duty, for it means that the night nurses available must wash and dress the patients, who are thus liable to be wakened too early in order that the work may be accomplished. Inattention to the food of nurses, as Dr. Menzies points out, is also a legitimate grievance, for in good private domestic service the dependants are better fed, and there is more variety, also their lives are better ordered than is the case in most of our public asylums.

I am not altogether in agreement with the notion advocated by Dr. Menzies of separate blocks for the nurses to live in. It is not natural for women to collect together in great flats, to and from which they trundle to meals and work in all weathers, and there is much to be said for the *sanctum* of a private bedroom adjacent to the nurse's duties to which, when tired or wishing to rest, she can conveniently retire.

There is much to be said for the so-called "club-room" for nurses being in a situation equally convenient for those of both sexes, and not in the "Nurses' Home," for the mingling of the sexes constitutes the best recreation, and it is the natural life for gregarious men and women. It is the separation of the sexes which asylum life insists upon and perpetuates that constitutes the chief reason for its unpopularity. Rooms of this kind, and collective entertainments for the staff which permit of social life, are undoubtedly more popular in asylums than when a lady presides over "small dances and sewing classes," and—"'twas ever thus"!

In public asylums, where most of the patients are of the poorer classes, the great curative agency is occupation, and the first rules of these institutions deal with employment, and by their example sensible young women of the better domestic service class, the daughters of small farmers and tradesmen, have rendered excellent service as nurses for the insane. An asylum population, under a good working staff, sees the routine of domestic and industrial work carried out by the nurses themselves, and such direct interest and effort dignifies labour and encourages the patients. The position of an asylum nurse is not a grateful one, for those patients who have recovered consider their incarceration—however excellent their care and humane their treatment—to be a stain upon their lives, and an unpleasant memory to revive, and the absence of this feeling of gratitude which recompenses nurses in general hospitals is distinctly hurtful to the *amour propre* of refined women, many of whom consider gratitude, and not a pecuniary equivalent, to be adequate reward for service to others. Moreover, the general *entourage* of a life spent among persons deficient in inhibition—and self-restraint is the characteristic feature of the higher man and woman—with its obvious consequences of bad language, abuse, personal violence, and excitement, is a stress and strain that educated ladies, the class aimed at, are unable to tolerate or even to discount, and it requires several years for men to do this.

Personally, I approve of the training encouraged by Dr. Menzies, but operations and surgical nursing, more especially of the nature of fractures, are not experiences which are welcomed in asylums. As to electro-therapeutics and massage, they are refinements rather than serious therapeutic remedies, and best attained elsewhere, and the resolution recently adopted by the Medico-Psychological Association to extend the period of training to three years, provided one is spent in a general hospital, seems to be a good solution. Moreover, now that every local authority is the responsible educational authority as well as the authority for dealing with public lunacy, it appears that these departments are best kept separate; and more benefit would, in my opinion, accrue from permitting the nurses to attend outside cookery demonstrations rather than in concocting creations or delicacies for unappreciative palates; for sane associations under such circumstances would be refreshing and recreative; moreover, criticism—the essence of progress—would be stimulating.

If Dr. Menzies asserts that for private patients of the upper classes, refined and educated ladies are necessary as companions and nurses, his suggestion will meet with cordial approval; but for the pauper classes in institutions, his views are open to criticism, and, moreover, they are not favoured by the results of his own practice.

Robert Jones.

### Everybody's Opinion.

[Correspondence on all subjects is invited, but we cannot in any way be responsible for the opinions expressed by our correspondents. No communication can be entertained if the name and address of the correspondent are not given as a guarantee of good faith, but not necessarily for publication. All correspondents should write on one side of the paper only.]

### THE CASE AGAINST CUBICLES.

The Chairman of Grimsby and District Hospital writes:— I have read your most interesting remarks on my letter of January 7th in The Hospital of this week. I consider your conclusions unanswerable, and I shall immediately lay your ideas before my committee with a view to having our plans altered at once. I thank you very much for your timely advice.

### NURSES VISITING BRIGHTON.

Miss E. H. Tompson and Miss McClellan write from 24 Charlotte Street, Brighton: For some time past a gathering for nurses has been held at the above address. Many of them have expressed much pleasure in spending an hour together and thus getting to know one another over the tea-table. You doubtless know how many strange nurses come to Brighton. Will you therefore kindly allow us to say, through you, how gladly we shall welcome, either resident nurses or those visiting Brighton temporarily. We trust that they will call upon us any Friday afternoon from 3.15 to 5 p.m.

### WOMEN ON HOSPITAL BOARDS.

Miss L. A. Jenner, 25 Warwick Gardens, Kensington, writes: Having just read the article with the above heading, which appeared in your issue of 3rd inst., and your own note appended to Lady Hermione Blackwood's letter on the same subject which appeared in that of 17th inst., I should be grateful if you could grant me space to touch on a few of the points raised. I gather in the first instance from the line taken that the writer of the article was not aware of the fact that, for years past, women, not a few, have been taking part, side by side with men, in the management of public institutions to the acknowledged advantage both of the work performed and of the inmates of the establishments. Surely women who have had such duties and powers entrusted to them again and again by those who are rated to supply the money which pays for the institutions, may be supposed in these matters hardly to deserve to be classed as mere "amateurs?" The example given of a "Ladies' Committee" which "interfered with the matron" and supported "the least worthy members of the nursing staff," cannot be regarded as being to the point, especially as you go on to state that what ultimately secured peace was the enactment of some drastic rules, which provided that no interference should take place "except by a resolution passed by a majority of the general committee which consisted largely of men." It would appear then that the general committee consisted of both men and women (presumably elected by, and responsible to, the fund-contributors), but that there was also a "Ladies' Committee," a body with power, but without responsibility—a dangerous condition of affairs wherever found! What is advocated by those who agree with the views set forth by Miss Georgiana Hill is that in the case of all hospitals in which women as well as men are admitted as patients, and in which women are employed as nurses, women as well as men should be eligible for direct election on to the boards of management. It is satisfactory to note that "The Hospital" "would be indisposed to support any hospital for incurables where the management does not provide for the admission, of women to its committees;" but it is difficult to understand from the reasons set forth for this conclusion why women should be more needed as members of the boards of management of hospitals for incurables than of those of hospitals whose patients are under treatment for the cure or alleviation of their diseases. It may interest you to know that, carrying on the work begun by her late father, Mr. George Hill, Miss Georgiana Hill has for several years been working, with the co-operation of some of the subscribers, to "bring about a reform in this matter" at the Royal Hospital for Incurables at Putney. Doubtless she will be glad of any assistance The Hospital can give towards the attainment of so desirable an end. In your note to Lady Hermione Blackwood's letter you say that you are convinced that no "true woman" will withhold her subscription to a well-administered voluntary hospital "because she is not elected to a seat on the Committee of Management." This seems to me to show a total misapprehension of the standpoint of those who agree with Miss Georgiana Hill. In the light of experience of women's work, public and private, they are convinced of the importance of the presence of directly-elected women, as well as men on the Boards of Management of hospitals, and the logical outcome of this is to support by subscriptions only such hospitals as fulfil this condition. The sick poor will lose nothing, for larger subscriptions can be sent to those institutions that have boards so constituted—in London, for example, to the Royal Free Hospital, Gray's Inn Road. May I, in conclusion, say that I for one entirely agree with the regret and dismay expressed by Lady Hermione Blackwood with reference to the attitude taken up by The Hospital on this subject.

[Our view is based upon wide experience of the administration and requirements of modern hospitals, where women are fully represented at present by the educated and capable ladies to be found on the staff in various capacities. In these institutions it is neither necessary nor desirable, for the reasons we have stated more than once before, that amateurs should be encouraged to interfere with the management, seeing that they possess neither technical knowledge nor any other special qualification to fit them for election.—Ed. The Hospital.]

### Appointments.

Denny Cottage Hospital.—Miss Agnes G. Small has been appointed nurse-matron. She was trained at the North Riding Infirmary, Middlesbro'-on-Tees, and has since been Queen's nurse, private nurse, and nurse-matron at Blairbridge School.

Nottingham Children's Hospital.—Miss Stanley has been appointed sister and Miss Marsh staff nurse. Miss Stanley was trained at St. Thomas's Hospital, London, and Miss Marsh at Poplar Hospital, London.

Hitchin Hospital.—Miss Mary Millhouse has been appointed head nurse. She was trained at Nottingham General Hospital, where she was afterwards staff nurse. She has also had training in district nursing and midwifery at Plaistow, and has been district nurse for three years at Hucknall, Yorkshire.

Isolation Hospital, Grenoside, Sheffield.—Miss Jeannie Brooks has been appointed nurse-matron. She was trained at Preston Royal Infirmary.

Lewes Workhouse Infirmary.—Miss H. Mand has been appointed charge nurse. She was trained at the Central Sick Asylum, Hendon, and has been staff nurse in the same institution.

Solihull Workhouse Infirmary.—Miss Frances Hannah Laws has been appointed superintendent nurse. She was trained at the Poplar and Stepney Sick Asylum, and has since been superintendent nurse at the Town Hospital, Guernsey.

Wolverhampton and Staffordshire General Hospital.—Miss Bertha L. Collins has been appointed sister of isolation ward. She was trained at Wolverhampton and Staffordshire General Hospital, and has since been nurse at Llandrindod Wells Hospital, night sister at West Suffolk Hospital, Bury St. Edmunds, and sister at the East Anglia Sanatorium, Nayland.

### Echoes from the Outside World.

### Movements of Royalty.

THE King and Queen concluded their visit to Chatsworth on Monday, the King returning to London, and the Queen, with Princess Victoria, proceeding to Sandringham. On Saturday their Majesties went to Buxton, and visited the Pavilion Gardens; they also inspected the pump-room and tasted the famous natural mineral waters. The town was profusely decorated, and the streets were thronged with spectators. The Queen called on Sunday at the village of Pilsley and offered her personal condolences to a widow—Mrs. Booth—whose son, one of the Chatsworth estate workmen, had died suddenly during the Royal visit.

On Tuesday the King paid a visit to Walmer Castle, and was conducted over the building, which he had not seen since he stayed there as a little boy with Queen Victoria as the guest of the Duke of Wellington.

### The Japanese in Possession of Port Arthur.

THE number of the surrendered garrison of Port Arthur is very much larger than was at first supposed. The total number of prisoners taken is 24,349, in addition to between 15,000 and 16,000 sick and wounded. There are 878 officers and 23,491 men, and it is stated that of the officers taken prisoners 50 per cent. will share the captivity of their men. Among the officers who have given their parole not to serve any more during the war is General Stössel, who will return home *viâ* Nagasaki. General Nogi states, in a report received at Tokio on Friday, that at the request of General Stössel he was pleased to meet the defender of Port Arthur at Shui-shi-ying on Thursday. The meeting lasted two hours and General Nogi, having through an interpreter expressed pleasure at meeting the general who had fought so bravely and gallantly for his Emperor and country, General Stössel thanked the Japanese commander for the pleasure of meeting his victorious army. General Nogi then stated that he had received a message from the Mikado asking that the greatest consideration should be shown to General Stössel and his officers in consideration of their splendid loyalty to their Emperor and country. Because of that wish, the Russian officers would be allowed to wear their swords. General Stössel expressed gratitude to the Mikado for thus saving the honour of his family. His descendants would appreciate the thoughtful kindness of the Mikado, and the same was true of his officers.

### Women and Agriculture.

THE Warden of Lady Warwick College, Studley Castle, Warwickshire (Miss Edith Bradley), gave an address last Friday afternoon at Caxton Hall, Westminster, on "The Lighter Branches of Agriculture as a most Promising Profession for Women." She said that the possibilities of employment for women trained in horticulture, dairying, and poultry and bee-keeping were becoming broader and wider year by year. The demand at present exceeded the supply, and posts offering good prospects had to be refused because a sufficient number of students did not train. They wanted people to look upon agriculture as a regular profession to be trained for, and seriously taken up. She proceeded to state that of the 146 fully-trained students who had passed through the College in less than eight years, about half were earning their own livelihood, or were in a position to do so. Small holdings were possessed by 24, and four more would shortly start; 12 were working in their home gardens or farms; 20 had obtained posts in England, and four in the Colonies, and six were on the College staff. The outlook for women, able to have their own holdings, was most promising. The chief difficulty was how to find the capital with which to start, and that, she thought, might be overcome by co-operation. The County Councils might render valuable help.

### Death of a French Revolutionist.

LOUISE MICHEL, who died at Marseilles on Monday in her 75th year, was a well-known figure during the riots and revolutionary movements which took place in France during the last days of the Third Empire. Her nickname was "The Red Virgin." Though she was a revolutionist and a communist of a most objectionable type, she was tender-hearted towards the poor, doing all in her power to help waifs and strays, and was always willing to share all she had with those less well off than herself. She at one time kept a school and gained some celebrity as a poetess, her verses being praised and accepted by Victor Hugo and Lamartine. In 1870 she was personally fighting against the Versailles troops and was dressed in the uniform of a national guard, being on one occasion wounded. She joined, if she did not herself organise, the band of women called the *pétroleuses* who saturated the furniture and floor of the Tuilleries Palace with petroleum and then set fire to it. In 1871 she was sentenced to transportation for life, but the amnesty of 1880 enabled her to return to Paris. In 1883 after lecturing in England, she was again imprisoned for inciting the people to pillage the bakers' shops.

### Disastrous Floods.

THE phenomenally high tide of Saturday last wrought great havoc in many parts of England. At Scarborough the north promenade pier, a structure built some years back, but lately acquired by the Mayor of Scarborough, and unfortunately uninsured, was swept away, only a portion of the pavilion and the entrance being left. No lives were lost, as the damage was done in the night. When daylight came, it was found that 800 feet of the 1,000 feet of which the pier was composed had completely disappeared. At Ramsgate the fish market was swamped, the esplanade flooded, and a quarter of a mile of it torn up. At Yarmouth the streets were flooded, so that the town had quite a Venetian appearance, and the beach gardens on which the unemployed have been at work were swept bare. At Spalding the water was 7 inches higher than the previous record, and even as far up the Thames as Putney, though it was not high tide till 3.45, at midday the river-bed was full, and there was not an appreciable ebb when the time came.

### Children at the Mansion House.

THE Lord and Lady Mayoress, as usual, celebrated Twelfth Night by holding the annual Juvenile Fancy Dress Ball. The number of children, exclusive of parents, exceeded 900, more being present than ever before. Among the guests who attracted attention were the Chinese Minister and his little girl in national costume, and the Crown Prince of Johore and his three little sons in Malay dress. These little boys, the eldest of whom appeared to be about seven or eight years of age, were immensely fascinated by the Punch and Judy show, and laughed delightedly when Toby seized Punch by the nose and shook him. Amongst noteworthy costumes were a tiny Cupid about four years old, with a wreath of crimson roses nestling in her dark curly hair; two little twin Robin Hoods; a Joan of Arc, whose dress of glittering chain-armour, with a white skirt, was one of the best in the room, the resemblance of the face and the mode of dressing the hair being wonderfully like the best-known portraits of the heroine; a diminutive Bishop, in all the glory of lawn sleeves; and an amusing representation of "Lobster Salad." A feature of the evening was the preponderance of Japanese dresses, both male and female.

### Notes and Queries.

**FOR REGULATIONS SEE PAGE 200.**

*Abroad.*

(112) 1. I should be very much obliged if you can tell me how to get nursing abroad. I have eight years' experience and can speak a little French. 2. Can you give me the name of the firm who advertised in THE HOSPITAL leather straps to attach a watch to the waist belt?—*Effie.*

1. Either by joining one of the nursing institutions abroad, or by advertisement. "The Nursing Profession, How and Where to Train," published by the Scientific Press, contains a list of Colonial and Foreign Nursing Institutions. 2. We believe that Mr. W. K. Stacy, 19 Newgate Street, E.C., sells what you require.

*Visiting.*

(113) Will you kindly inform me which is the best way to begin visiting nursing? What fees should I charge? If circulars are necessary, how should they be worded?—*A. C.*

You should send cards with name, qualification and terms to all the medical men in the vicinity in which you propose working. The terms usually range from 3s. 6d. an hour.

*Midwifery.*

(114) Can you kindly tell me where I could sit for my midwifery certificate without expense or loss of time? I was trained by my aunt, who was formerly on the Ext. Department at Queen Charlotte's Hospital, and I have been acting as midwife for several years.—*Nurse G.*

Apply for registration to the Central Midwives Board, 6 Suffolk Street, Pall Mall.

A nurse trained in 1898 has just failed to get her L.O.S. Will this necessitate her resigning her country district?—*Uniform.*

Write for advice to the Midwives Institute, 12 Buckingham Street, Strand, W.C.

Will you kindly tell me if I can continue my work as monthly nurse after the Midwives Act comes into force? I do not possess the L.O.S. certificate.—*Swallow.*

The Act does not relate to, nor interfere with, monthly nurses.

Will you kindly inform me how I can obtain a post as district midwife?—*Nurse Anne.*

Advertise and reply to advertisements. Also write to the Association for Promoting the Training and Supply of Midwives, Dacre House, New Tothill Street, Westminster, S.W.

*Midwifery Training.*

(115) Will you kindly send me full particulars and fees for midwifery training?—*Jessie W.*

Write to the Midwives Institute, 12 Buckingham Street, Strand, W.C.

*Ship's Nurses.*

(116) Can you kindly tell me of any line of steamers which carry trained nurses as stewardesses?—*Nurse P.*

Trained nurses are not yet employed as stewardesses.

*Home.*

(117) Can you tell me of a home where a poor and helpless woman suffering from rheumatism could be received?—She can only pay a small sum per week.—*Miss K.*

You do not give sufficient particulars; a great deal depends upon the amount you can pay. A full list of Homes is given in "Burdett's Hospitals and Charities."

*Male Nurse.*

(118) Can you kindly tell me if nurses (male) trained in Britain would be recognised in America? What qualifications are necessary in order to be registered there?—*A Male Nurse.*

Yes. You must apply for particulars of the association you propose to join.

*Home.*

(119) *Nurse W.* would be much obliged for any information of homes for nurses unable to work any longer.

Write to the Secretary of the Royal United Nurses Association, 10 Orchard Street, W.

**Handbooks for Nurses.**

| | Post Free. |
|---|---|
| How to Become a Nurse: "The Nursing Profession" ... | 2s. 4d. |
| "Nurses' Pronouncing Dictionary." Terms and Treatment... | 2s. 0d. |
| "A Handbook for Nurses." Dr J. K. Watson ... | 5s. 4d. |
| "Elementary Physiology for Nurses" ... | 2s. 0d. |
| "Swedish Massage and Movements" ... | 2s. 6d. |

Of all booksellers or of The Scientific Press, Limited, 28 & 29 Southampton Street, Strand, London, W.C.

### For Reading to the Sick.

"HE THAT ENDURETH TO THE END SHALL BE SAVED."

HOPE still; though darkness round thee spread,
Count mercy in the clouds o'erhead,
And lean upon thy God.
Wait for the strength the Lord will send:
He that endureth to the end
Shall win the crown at last;
Nor will he mourn the way was dim;
Christ trod a darker way for him,
And clasps his weak hand now.

*A. Shipton.*

The late Dean Goulburn says in his *Thoughts on Personal Religion* (Part iii., chap. 9): "It is necessary that suffering be regarded as a vocation."

There are many people who think it an interference with their vocation, and who consequently miss all its golden opportunities of growth in grace and knowledge.

"You must familiarise yourself with the idea, that through sickness your occupation is not gone, but that it is simply altered." No one can be perfect who has not submitted to the discipline of trial, of suffering. No one is wholly like Christ who has not learnt to endure, even unto death. "If any man will come after Me, let him deny himself, and take up his cross and follow Me."—Matt. xvi., 24.—*E. A. D.*

Thy cross is quite enough for thee.
Though little it appears;
For there is hid in it the weight
Of the eternal years.

He practises all virtues well
Who his own cross reveres,
And lives in the familiar thought
Of the eternal years.

*F. W. Faber.*

How hard it is for some of us to be put aside, to be obliged to give up this and that labour of love, this or that duty which has been ours! how difficult to be idle, to be glad of opportunities for quiet meditation and thought, and to rejoice that we are called to suffer with Christ!

There is only one way to succeed in repressing our natural unwillingness, and that is to fix our thoughts entirely away from ourselves, and so to fill ourselves with thoughts of Christ and what He bare for us that there is no room in our minds or hearts for any grumbling or repining thoughts.

Let us bear our cross with Him, and follow meekly, like Simon the Cyrenian, in His blessed footsteps, even though He lead us past our Calvary to Heaven.—*E. A. D.*

Gird up thyself, and come and stand
Unflinching under the unfaltering Hand,
That waits to prove thee to the uttermost!
It were not hard to suffer by His hand,
If thou could'st see His face—but in the dark!
That is the one last trial—be it so.
Christ was forsaken, so must thou be too;
How could'st thou suffer but in seeming, else?

*H. Hamilton King.*

